# Association between lower limb strength, anxiety, and depression symptoms in older adults

**DOI:** 10.1590/1806-9282.20260070

**Published:** 2026-08-07

**Authors:** Poliany Pereira Cruz, Ricardo Augusto Leoni De Sousa, Laís Francielle Francisca Felício, Renato Sobral Monteiro-Junior, Ricardo Cardoso Cassilhas

**Affiliations:** 1 Universidade Federal dos Vales do Jequitinhonha e Mucuri, Neuroscience and Exercise Study Group (Grupo de Estudos em Neurociências e Exercício – GENE) – Diamantina (MG), Brazil.; 2 Universidade Federal dos Vales do Jequitinhonha e Mucuri, Post Graduate Program in Health Sciences – Diamantina (MG), Brazil.; 3 Wright State University, School of Health and Exercise Sciences – Dayton (OH), United States.; 4 Universidade Federal dos Vales do Jequitinhonha e Mucuri, School of Biological and Health Sciences, Department of Physical Education – Diamantina (MG), Brazil.; 5 Universidade Estadual de Montes Claros, Post Graduate Program in Health Sciences – Montes Claros (MG), Brazil.

**Keywords:** Older adults, Muscle strength, Anxiety, Depression, Mental health

## Abstract

**OBJECTIVE:**

The aim of this study was to evaluate the association between lower limb strength and symptoms of anxiety and depression in communitydwelling older adults.

**METHODS:**

This cross-sectional study included 99 community-dwelling older adults (69.5±6.6 years; 81.8% women) assessed between 2021 and 2023 in Minas Gerais, Brazil. Inclusion criteria were age ≥60 years, independent mobility, and medical clearance. Participants were excluded if they had uncontrolled chronic or cardiovascular diseases, a history of stroke, unmanaged neurodegenerative disorders, active psychiatric comorbidities that could interfere with psychological assessments, or musculoskeletal injuries preventing safe participation in testing. Anxiety and depressive symptoms were assessed using the Geriatric Anxiety Inventory and Geriatric Depression Scale. Lower limb strength was measured using the 30-Second Chair Stand test and classified according to age- and sex-specific normative values.

**RESULTS:**

Participants with below-average lower limb strength had higher odds of anxiety symptoms (OR 10.90; 95%CI 1.97–60.33; p=0.006). Lower limb strength was also associated with depressive symptoms, although with a smaller effect size (OR 3.96; 95%CI 1.06–14.74; p=0.040). Age was positively associated with anxiety (p=0.019), while no significant associations were observed for sex.

**CONCLUSION:**

The 30-Second Chair Stand test may have potential as a complementary screening indicator to identify older adults at elevated psychosocial risk.

## INTRODUCTION

The global population aging shifts the focus of discussion from lifespan to quality of life^1^. Healthy Life Expectancy indicates that a portion of the additional years gained may be lived with health limitations, reinforcing the importance of maintaining functional capacity^2^. For example, sarcopenia is one of the main conditions associated with functional decline in aging. Sarcopenia is a progressive disorder characterized by the loss of muscle mass and strength, which are associated with an increased risk of falls, disability, and mortality^3^. In this context, strategies that promote autonomy and reduce the duration of life lived with disability across the lifespan are urgently needed. Therefore, muscle strength is a key determinant of functional capacity, supporting mobility, balance, and self-care^4^.

Muscle strength decline is linked to poorer performance in activities of daily living and an increased risk of adverse events^5^. Within this framework, lower limb strength (LLS) is particularly relevant, as it directly reflects the demands of locomotion, such as walking, rising from a chair, climbing stairs, and is associated with a higher risk of falls when reduced^6^. From a psychosocial perspective, lower levels of muscle strength have been associated with a higher prevalence of anxiety and depressive symptoms in adult and older populations^7^. This relationship is possibly mediated by a cycle of disuse, involving difficulties with basic tasks, social isolation, and reduced self-esteem.

However, a gap remains in the literature as most studies use handgrip strength as a marker of overall muscle strength^8^, and, therefore, neglecting the specific role of LLS. Recent evidence, however, suggests that LLS may show a stronger association with psychological outcomes. In adjusted analyses, knee extension strength has demonstrated a more robust relationship with depressive symptoms, as it more directly reflects locomotor capacity and social participation in older adulthood^9^. Therefore, it is crucial to develop guiding strategies to promote mental health and functional capacity in older adulthood. The aim of the present study was to investigate the association between LLS and the presence of anxiety and depressive symptoms in community-dwelling older adults.

## METHODS

This study was approved by the Research Ethics Committee of Universidade Estadual de Montes Claros, approval number 2,741,071, and conducted in accordance with the Declaration of Helsinki. During the COVID-19 pandemic, preventive measures were implemented, including mask use, 70% alcohol hand hygiene, room ventilation, and temporary exclusion of symptomatic individuals. For activities involving physical exercise, intensity was monitored using the Rating of Perceived Exertion scale^10^.

### Study design

This was an observational, cross-sectional study done in the state of Minas Gerais, Brazil. Eligible participants were assessed between 2021 and 2023. Inclusion criteria involved individuals aged ≥60 years, with medical clearance for participation, independent mobility (with or without assistive devices), and the ability to communicate were eligible. Exclusion criteria included uncontrolled chronic or cardiovascular diseases, prior stroke, unmanaged neurodegenerative disorders, active psychiatric comorbidities that could interfere with psychological assessments, or musculoskeletal injuries preventing safe participation in the tests. A total of 102 volunteers were assessed, and 3 were excluded based on clinical criteria (1 stroke, 1 Parkinson’s disease, and 1 Alzheimer’s disease), resulting in 99 participants included in the study. Finally, a total of 99 older adults were included (mean age 69.5±6.6 years), with a predominance of females (81.8%).

Data collection was carried out by a previously trained multidisciplinary team. A standardized interview gathered sociodemographic and clinical information from participants, including age, sex, education, marital status, body mass index, and physical activity. Depressive symptoms were assessed using the Geriatric Depression Scale—15 items (GDS-15). A cutoff score of 5 was adopted (≥5 indicating the presence of depressive symptoms). Anxiety symptoms were assessed using the Geriatric Anxiety Inventory (GAI). A cutoff score of 13 was adopted (≥13 indicating the presence of anxiety symptoms). LLS was assessed using the 30-Second Chair Stand test (CS-30), as previously described. The number of repetitions completed in 30 s was recorded as a continuous measure of LLS. For descriptive and analytical purposes, LLS was also categorized according to age- and sex-specific normative ranges.

### Statistical analysis

Analyses were conducted using IBM SPSS Statistics 24.0 for the tables and GraphPad Prism 8 for the figure. For each dichotomized outcome, anxiety (GAI ≥13 vs. <13) and depression (GDS-15 ≥5 vs. <5), binary logistic regression models were estimated. A parsimonious model approach was pre-specified due to the limited number of events per variable. Odds ratios (ORs) with 95%CIs and two-tailed p-values were estimated. Model fit was assessed using the Hosmer-Lemeshow test, and Nagelkerke R^2^, model χ^2^, and the number of cases included (listwise) were reported. Cases with missing data for variables of interest were excluded on a case-wise basis.

## RESULTS

The sociodemographic and clinical characteristics of the sample are shown below (Table 1).

**Table 1:** Sample characteristics (n=99).

Characteristic	Category	n	Valid %
Age (mean±SD)	–	69.5±6.6	–
Sex	Female	81	81.8
	Male	18	18.2
Education	0 years	3	3.1
	1–4 years	22	22.4
	5–8 years	11	11.2
	9–12 years	41	41.8
	13+ years	21	21.5
Marital status	Single	11	11.3
	Married	45	46.4
	Widowed	28	28.9
	Divorced	13	13.4
Body mass index	Normal weight	28	32.2
	Overweight	34	39.1
	Obesity	25	28.7
Race/ethnicity (self-reported, IBGE)	White	28	34.6
	Brown	41	50.6
	Black	11	13.6
	Asian	1	1.2
Religion	Catholic	61	74.4
	Evangelical	19	23.2
	Agnostic	1	1.2
Regular physical activity	No	44	44.4
	Yes	55	55.6

SD: standard deviation.

In the adjusted models (Table 2), participants with below-average CS-30 performance had 10.90 times higher odds of anxiety symptoms compared to those with normal/above-average performance (OR 10.90; 95%CI 1.97–60.33; p=0.006). Age was positively associated with anxiety (OR 1.18 per year; 95%CI 1.03–1.36; p=0.019), whereas sex was not significantly associated (p=0.145). For depressive symptoms, although the overall model was marginally significant, participants with below-average performance had 3.96 times higher odds of depressive symptoms compared to those with normal/above-average performance (OR 3.96; 95%CI 1.06–14.74; p=0.040). Age (OR 1.05; 95%CI 0.96–1.15; p=0.323) and sex (OR 2.22; 95%CI 0.49–9.98; p=0.300) were not significantly associated with depressive symptoms.

**Table 2:** Association between lower limb strength (30-Second Chair Stand test), anxiety, and depressive symptoms.

Variable	Adjusted OR	95%CI	p
Anxiety (GAI ≥13), n=74			
LLS—below vs. normal/above	10.90	1.97–60.33	0.006
Age (per year)	1.18	1.03–1.36	0.019
Sex (male=1)	5.50	0.55–54.60	0.145
Depression (GDS-15 ≥5), n=59			
LLS—below vs. normal/above	3.96	1.06–14.74	0.040
Age (per year)	1.05	0.96–1.15	0.323
Sex (male=1)	2.22	0.49–9.98	0.300

OR: odds ratio; CI: confidence interval; GAI: Geriatric Anxiety Inventory; LLS: lower limb strength; GDS-15: Geriatric Depression Scale—15 items.

Model fit indices indicated that the logistic regression model for anxiety showed good fit [χ^2^(3)=21.884; Hosmer-Lemeshow p=0.813; Nagelkerke R^2^=0.423; n=74], while the model for depression demonstrated an acceptable fit [χ^2^(3)=7.054; Hosmer-Lemeshow p=0.644; Nagelkerke R^2^=0.159; n=59]. OR with 95%CI are presented. LLS, assessed by the CS-30, was categorized according to age- and sex-specific normative ranges.

**Figure 1:**
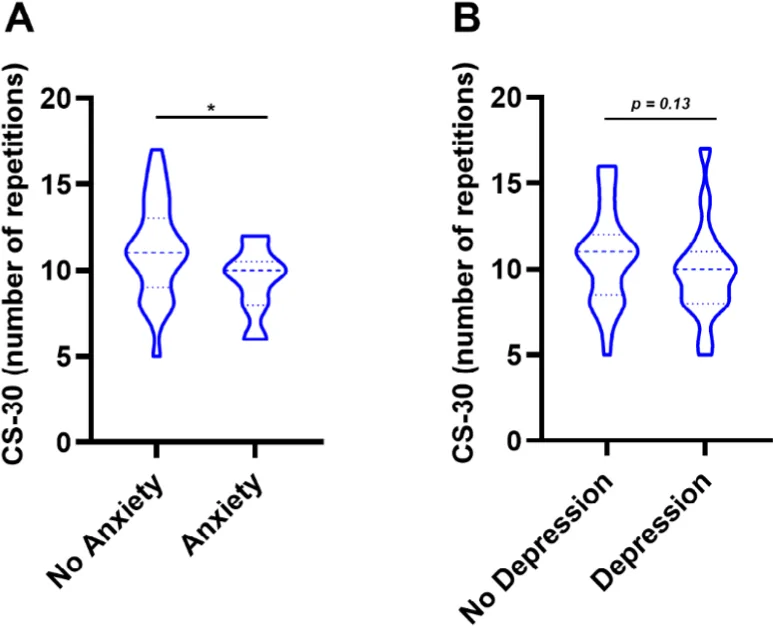
Distribution of 30-Second Chair Stand test performance according to the presence of anxiety and depressive symptoms. Violin plots illustrate the distribution, median, and interquartile range of 30-Second Chair Stand test scores for participants with and without clinically significant anxiety (A) and depressive symptoms (B). Lower 30-Second Chair Stand test performance was significantly associated with anxiety (*p<0.05), whereas the difference for depressive symptoms showed only a marginal trend (p=0.13). These unadjusted group differences visually complement the logistic regression findings reported in Table 3.

Thereafter, we did the violin-plot comparisons to provide a visual representation of the raw CS-30 distributions. As expected, these unadjusted comparisons yielded a significant difference for anxiety but only a tendency for depression (p=0.13), which is consistent with the weaker and borderline-significant associations observed in the logistic regression models (Figure 1).

**Table 3:** Association between lower limb strength (30-Second Chair Stand test) and anxiety and depressive symptoms.

Variable	Adjusted OR	95%CI	p
Anxiety (GAI≥13), n=74			
LLS—Below vs. normal/above	10.90	1.97–60.33	0.006
Age (per year)	1.18	1.03–1.36	0.019
Sex (male=1)	5.50	0.55–54.60	0.145
Depression (GDS-15≥5), n=59			
LLS—Below vs. normal/above	3.96	1.06–14.74	0.040
Age (per year)	1.05	0.96–1.15	0.323
Sex (male=1)	2.22	0.49–9.98	0.300

OR: odds ratio; CI: confidence interval; GAI: Geriatric Anxiety Inventory; LLS: lower limb strength; GDS-15: Geriatric Depression Scale—15 items.

## DISCUSSION

In community-dwelling older adults, LLS assessed by the CS-30 was inversely associated with symptoms of anxiety and, to a lesser extent, depression. These findings suggest that reduced LLS may contribute to increased psychosocial vulnerability in later life, highlighting the potential role of muscular function in maintaining mental health. The violin plots further supported these findings by illustrating significantly lower CS-30 scores in participants with anxiety, while a non-significant trend was observed for depression which was consistent with the weaker association detected in the regression models. The association appeared stronger for anxiety, indicating that lower extremity weakness may have a particularly relevant impact on anxious symptomatology. These findings add to the existing observational literature reporting an association between muscle strength and mental health^7,11,12^.

A key strength of the present study was its focus on LLS, assessed using a functional and normative test (CS-30), which enhances the understanding of the relationship between physical performance and psychological symptoms in community-dwelling older adults. From a practical perspective, our findings carry immediate clinical implications as simple screenings of LLS, such as the CS-30, may serve as valuable tools in primary care and community settings. Given its feasibility and low cost, this assessment can help rapidly identify older adults at higher risk of anxiety and depressive symptoms, guiding referrals to multicomponent interventions, including LLS programs.

There is biological plausibility for the link between greater LLS and better mental health^12,13^. LLS reflects neurobiological adaptations associated with exercise, particularly resistance training, such as increased levels of brain-derived neurotrophic factor and insulin-like growth factor 1^14^, modulation of the hypothalamic–pituitary–adrenal axis^15^, and regulation of mood-related neurotransmitters, in addition to central anti-inflammatory effects^16^. These mechanisms provide a plausible explanation for the observed association between greater LLS and a lower likelihood of anxiety and depressive symptoms, while also promoting mobility, autonomy, and social participation. However, the present study did not quantify physical activity. Therefore, these mechanisms are inferred and do not imply causality.

Beyond human observational evidence, translational studies using animal models may help clarify the mechanistic pathways through which improved LLS influences mental health and cognitive function^17^. Experimental resistance-training paradigms in rodents provide a controlled platform to test causal neurobiological effects that cannot be directly examined in older adults. For example, a recent study using a high-intensity resistance-training protocol in a rat model of type 2 diabetes demonstrated that strength training preserved cognitive performance and attenuated neurobiological markers of brain insulin resistance, specifically by increasing hippocampal levels of insulin receptor substrate-1 and inhibiting the activation of glycogen synthase kinase-3 beta, a kinase implicated in neuronal injury and Alzheimer-related pathology^18^. Although these animals were not aged, the findings reveal that improvements in muscular strength can modulate insulin-signaling pathways in the hippocampus, a region central to memory, emotional regulation, and vulnerability to stress responses.

Finally, it is important to acknowledge that the wide confidence intervals observed in some of the regression estimates suggest limited precision and potential model instability, likely related to the sample size and number of outcome events. Therefore, these findings should be interpreted with caution.

## CONCLUSION

Our findings highlight the potential of simple, low-cost functional assessments to identify older adults at higher psychosocial risk and underscore the importance of maintaining LLS for both physical and mental health. While causality cannot be inferred, our results provide a strong rationale for translational and longitudinal studies, and clinical trials targeting LLS as a strategy to promote mental well-being, functional independence, and overall healthy aging.

## Data Availability

The datasets generated and/or analyzed during the current study are available from the corresponding author upon reasonable request.
